# Methodological Factors Affecting Canine Vaginal Cytology: The Influence of Sampling Site, Swab Condition, and Fixation Method

**DOI:** 10.1155/vmi/6663541

**Published:** 2026-06-30

**Authors:** Katrin von Plato, Sait Sendag, Mehmet Yildiz, Axel Wehrend

**Affiliations:** ^1^ Veterinary Clinic for Reproductive Medicine and Neonatology, Justus Liebig-University, Giessen, Germany; ^2^ Department of Obstetrics and Gynecology, Faculty of Veterinary Medicine, Van Yuzuncu Yil University, Van, Türkiye, yyu.edu.tr

**Keywords:** bitch, estrous cycle, methodological standardization, vaginal cytology

## Abstract

**Background:**

Vaginal cytology is widely used for cycle staging in bitches because of its practicality and low cost; however, its diagnostic reliability is strongly influenced by methodological variability. The present study aimed to evaluate the impact of sampling site, swab type, and fixation method on the interpretation of exfoliative vaginal cytology.

**Materials and Methods:**

Exfoliative vaginal cytology samples were obtained from 332 clinically healthy bitches representing all stages of the estrous cycle. Samples were collected from different locations (vestibulum vs. vagina) using either dry or saline‐moistened swabs and prepared as air‐dried or spray‐fixed smears, followed by eosin–thiazine staining. Clinical examination and serum progesterone concentrations served as the reference method for cycle staging.

**Results:**

The proportions of different cell types were significantly influenced by the sampling location. Vestibular smears contained higher proportions of keratinized and intermediate cells (*p* < 0.0001), whereas basal cells (*p* = 0.0078) and anuclear superficial cells (*p* < 0.0001) were more frequently observed in vaginal smears. Overall, sampling location significantly affected the distinction between proestrus and estrus. Swab condition significantly influenced smear quality and cellular presentation but had minimal impact on cycle stage classification. Smears obtained using saline‐moistened swabs showed significantly higher cellular yield per microscopic field (*p* < 0.0001). Bacteria, cellular debris, and secretory material were less frequently observed in moistened smears (*p* < 0.0001), whereas free nuclei were detected more frequently (*p* < 0.0001). Cotton fibers were more common in dry swab preparations (*p* < 0.0001). Cellular arrangement also differed between sampling methods (*p* = 0.003). The fixation method influenced certain morphological features but did not affect cytological stage classification. No significant differences in total cellular yield were observed between air‐dried and spray‐fixed smears.

**Conclusion:**

Among the factors evaluated, only the sampling location exerted a clinically relevant effect on cycle stage determination, particularly for distinguishing proestrus from estrus.

## 1. Introduction

In canine reproduction, particularly within the context of controlled breeding programs, precise diagnosis of estrous stages has become increasingly important. In modern breeding practice, bitches are frequently transported over long distances to selected stud dogs. Accurate prediction of the optimal mating time is therefore essential to ensure successful fertilization and to maximize litter size. This precision is equally critical in artificial insemination programs, where extended storage, particularly cryopreservation, may reduce sperm fertilizing capacity, thereby narrowing the fertile window and increasing the demand for exact cycle staging [1, 2]. Vaginal cytology is one of the most commonly applied methods for determining the reproductive stage in bitches and serves as a practical tool to narrow the timing of serum progesterone sampling; however, it does not replace progesterone assay, particularly when precise ovulation timing is required, such as for breeding with frozen semen. The exfoliative cellular changes of the vaginal epithelium closely reflect underlying hormonal dynamics and allow reliable differentiation among proestrus, estrus, and early diestrus. However, mid‐to late diestrus may present cytological features similar to anestrus, in which case serum progesterone measurement is required for accurate differentiation of cycle stages. As a result, vaginal cytology has attracted considerable attention and is extensively described in the veterinary literature [1, 3–7]. Despite its widespread application, vaginal cytology in canine reproduction is characterized by substantial methodological and interpretative heterogeneity. In contrast to human gynecology, where collection, handling, and evaluation of cytological samples from the female genital tract are highly standardized in order to prevent misdiagnosis [8], no universally accepted guidelines exist in canine reproduction medicine. Variations in sampling techniques, smear preparation, fixation methods, staining protocols, and evaluation criteria are therefore common. This lack of standardization has resulted in notable inconsistencies in published descriptions and diagnostic interpretations of vaginal cytology [9, 10]. Notably, despite the extensive literature on the subject, there is a paucity of studies specifically addressing the reproducibility and diagnostic reliability of individual cytological methods. The absence of comparative evaluations, including assessments of observer‐dependent variability, further limits the clinical robustness of vaginal cytology, particularly in teaching environments. Although endocrine diagnostics, such as serum progesterone measurement, provide the objective reference standard for reproductive staging and optimal breeding time determination, their routine application may be constrained by economic, logistical, or temporal factors. In this context, vaginal cytology serves as a valuable adjunctive tool that helps identify the transition from proestrus to estrus and thereby reduces the number and improves the timing of blood collections for progesterone assays, without replacing endocrine confirmation. Consequently, vaginal cytology remains a cornerstone of reproductive diagnostics in daily clinical practice, underscoring the need for standardized, reproducible, and easily applicable protocols. The present study was therefore designed to critically examine methodological determinants influencing vaginal cytology in bitches by evaluating the effects of sampling site, swab condition, and fixation method on cytological characteristics and estrous cycle classification. By benchmarking cytological findings against progesterone‐based hormonal staging, this study aims to identify procedural factors that most strongly influence diagnostic reliability and to contribute to the establishment of standardized and reproducible cytological protocols in canine reproduction.

## 2. Materials and Methods

### 2.1. Ethical Approval

All examinations and procedures were performed as part of routine clinical reproductive management and good veterinary clinical practice in bitches presented to the clinic for determination of the optimal mating time, estrous cycle staging, or preneutering evaluation. No procedures were performed solely for research purposes, and no additional blood samples, swabs, or other diagnostic interventions beyond standard clinical management were obtained for this study. All clinical data were derived exclusively from routine diagnostic procedures, and no additional discomfort, risk, or invasive intervention was imposed on the animals. Informed owner consent for the use of anonymized clinical findings and data for scientific publication was obtained in all cases prior to inclusion in the study. All procedures were conducted in accordance with institutional and national guidelines for veterinary clinical practice and animal welfare. As no experimental interventions beyond routine clinical diagnostics and management were performed, approval by an Institutional Animal Care and Use Committee was not required.

### 2.2. Animals

The study included 332 clinically healthy bitches presented to our reproductive medicine clinic (Veterinary Clinic for Reproductive Medicine and Neonatology, Justus Liebig University, Giessen, Germany) for routine reproductive evaluation. Animals ranged in age from 1 to 13 years (mean ± SD: 4.52 ± 2.58 years) and represented various breeds. All examinations and sample collections were performed exclusively as part of routine reproductive diagnostic procedures.

### 2.3. Determination of Cycle Stage

Cycle stage was determined using an integrated diagnostic approach combining anamnestic data, behavioral observations, clinical examination, vaginoscopic findings obtained using a rigid endoscope (Karl Storz, Germany), vaginal cytology, and plasma progesterone concentrations. Cytological findings characteristic of the different phases of the estrous cycle were interpreted in accordance with the classification proposed by Reckers et al. [10]. Vaginal cytology was evaluated based on established cytomorphological criteria. The cycle stages are defined as follows.

Proestrus: Presence of hemorrhagic vaginal discharge and progressive vulvar edema, accompanied by increasing mucosal folding, with plasma progesterone concentrations < 2.0 ng/mL. During proestrus, the proportion of epithelial cells with abundant and polygonal‐shaped cytoplasm progressively increases in vaginal smears. In the majority of bitches, erythrocytes are commonly observed throughout this phase.

Estrus: Pronounced vulvar edema; serosanguinous vaginal discharge may be present; and pale, dry, and highly folded vaginal mucosa, typically associated with plasma progesterone concentrations ranging from 2.0 to 15.0 ng/mL [4], although higher values may also occur in some bitches during estrus. These criteria were not required to be present simultaneously; final cycle stage classification was based on an integrated assessment of endocrine values, clinical findings, behavioral acceptance, and the dominant cytological cell population. Cytologically, the smear is dominated by epithelial cells with abundant and polygonal‐shaped cytoplasm, which are predominantly anuclear. An estrous cytological pattern is defined when more than 90% of the cells present are superficial cells or anuclear squamous cells. At the time of ovulation, epithelial cells frequently appear in clusters (cell aggregation).

Early diestrus: Period within the first 30 days after the end of estrus, characterized by the absence or progressive resolution of vulvar edema and vaginal discharge, with plasma progesterone concentrations typically > 15 ng/mL. In the early phase, neutrophilic granulocytes appear in increased numbers. Vaginal smears are predominantly composed of intermediate and parabasal cells. During the first days of early diestrus, a mixed cell population may still be observed, with occasional residual superficial cells.

Anestrus: Period between 60 and 100 days after the end of estrus, characterized by a nonedematous vulva, absence of vaginal discharge, and a moist, pale pink vaginal mucosa without mucosal folding, with plasma progesterone concentrations < 1.5 ng/mL. Cytologically, smears contain predominantly parabasal and intermediate cells. Overall cellularity is low during this phase.

These cytological criteria were applied consistently across all samples to ensure standardized classification of estrous cycle stages. Final cycle stage classification was established based on the combined findings by a diplomate of the European College of Animal Reproduction (ECAR).

### 2.4. Experimental Design

To assess the influence of methodological variables on cytological evaluation, three experimental comparisons were conducted: Experiment I assessed the effect of sampling site by comparing cytological samples obtained from the cranial vagina and the vestibulum using dry cotton swabs (*n* = 120 bitches). Experiment II investigated the influence of swab condition by comparing dry and saline‐moistened cotton swabs for vaginal sampling (*n* = 106 bitches). Experiment III examined the impact of fixation technique by comparing air‐dried and spray‐fixed smears (*n* = 106 bitches).

### 2.5. Vaginal Cytology Sampling

After gentle cleansing of the vulvar area, two paired samples were collected from each bitch in a fixed sequence, first from the cranial vagina and subsequently from the vestibulum. In both cases, a sterile cotton swab (Heinz Herenz GmbH, Hamburg, Germany; 150 mm in length, 4 mm in shaft diameter, with a 10 mm cotton tip diameter) was introduced through a tubular vaginal speculum without contact with the labia, using either dry or saline‐moistened swabs depending on the experimental group. Samples were obtained using gentle rotational movements. Immediately after collection, the swabs were rolled onto degreased glass slides (76 × 26 mm, iDL, Nidderau, Germany) using three parallel strokes.

### 2.6. Smear Preparation and Staining

Immediately after sampling, each individual sample (vaginal or vestibular) was rolled onto its own separate degreased glass slide (76 × 26 mm; iDL, Nidderau, Germany) to create three parallel smear tracks. Depending on the experimental group, smears were either air‐dried or immediately spray‐fixed. Air‐drying was performed for a minimum of 5 min under ambient laboratory conditions to ensure complete drying, particularly in smears obtained using saline‐moistened swabs. For the fixation experiment, two separate vaginal samples were collected from the dorsal vaginal wall using individual dry cotton swabs. One smear was left to air‐dry for 4 days, whereas the second smear was immediately spray‐fixed after preparation from a distance of approximately 20 cm using a commercial cytology fixative (Merckofix, Merck, Darmstadt, Germany). After air‐drying or fixation, all slides were stained using a rapid eosin–thiazine staining method (Hämacolor, Merck, Darmstadt, Germany) according to the manufacturer’s instructions and permanently mounted with coverslips using a synthetic mounting medium (Entellan, Merck, Darmstadt, Germany).

### 2.7. Endocrinological Analysis

Venous blood samples were collected for the determination of plasma progesterone concentrations. Progesterone levels were measured using an enzyme‐linked fluorescent assay (mini VIDAS, bioMérieux, France), which has been validated for use in bitches [11].

### 2.8. Cytological Evaluation

Cytological examination and morphometric assessment were performed using a light microscope (DMR, Leica Microsystems, Wetzlar, Germany) equipped with a digital camera. Images were transmitted in real time to a computer and documented using image analysis software (Leica Image Manager, Leica Microsystems, Wetzlar, Germany). Smears were initially screened at 100 × magnification to assess overall quality. Slides exhibiting insufficient cellularity, severe staining artifacts, or pronounced sampling‐related artifacts were excluded from further analysis. Quantitative cytological evaluation was conducted at 400 × magnification. For each smear, 200 epithelial cells were counted across at least four randomly selected microscopic fields to ensure representative sampling. Epithelial cells were classified according to standardized cytomorphological criteria into basal, parabasal, intermediate, superficial, anuclear superficial (cornified squames), and highly keratinized (KZ) superficial cells. In the present study, the latter term refers to advanced end‐stage morphologic forms of superficial epithelial cells characterized by marked keratinization, flattened angular morphology, and absence of a visible nucleus, rather than representing a distinct cytological cell type.

The defining morphological characteristics used for cell identification and differentiation are summarized in Table 1, which provides the cytological classification framework underlying all quantitative analyses in this study. Cellular dimensions listed in Table 1 were determined by light microscopic morphometric analysis using a DMR light microscope (Leica Microsystems, Wetzlar, Germany) connected to a digital camera and image analysis software (Leica Image Manager, Leica, Wetzlar, Germany). All cytological evaluations were performed by a single veterinarian specializing in small animal reproduction. For each paired sample, each smear was independently assigned to an estrous cycle stage based on its cytological characteristics and subsequently classified as concordant or discordant with the reference diagnosis. Cellular yield (cellularity) per microscopic field was assessed by counting a total of 200 epithelial cells per smear at 400× magnification, distributed across at least four representative microscopic fields selected in a meander‐shaped scanning pattern. Cell classification was based on the morphological criteria summarized in Table 1.

**TABLE 1:** Morphological characteristics features of epithelial cell types used in cytological evaluation.

Epithelial cell type	Morphological characteristics
Basal cells	10–20 μm; round to ovoid; smooth cell margins; narrow cytoplasmic rim; eccentrically located, spherical nucleus
Parabasal cells	15–25 μm; smooth cell borders; clearly visible centrally positioned nucleus; distinct cytoplasmic rim (Figure 1A)
Intermediate cells	20–35 μm; round to oval; smooth margins; distinct nucleus; cytoplasm‐to‐nucleus ratio shifted toward cytoplasm; frequently showing the characteristic “fried‐egg” appearance (Figure 1B)
Superficial cells	42–58 μm; polygonal to angular shape; often folded or irregular cell margins; abundant clear cytoplasm; nucleus still visible but typically pyknotic (Figure 1C)
Anuclear superficial cells (cornified squames)	42–58 μm; morphologically similar to superficial cells but lacking a visible nucleus (Figure 1D)
Highly keratinized (KZ) superficial cells	Advanced end‐stage morphologic form of superficial epithelial cells; intensely eosinophilic cytoplasm; absent nucleus; angular to irregular cell borders; flattened, polygonal, or slightly folded morphology; representing highly keratinized variants of superficial and anuclear squamous cells (Figure 1F)

**FIGURE 1:**
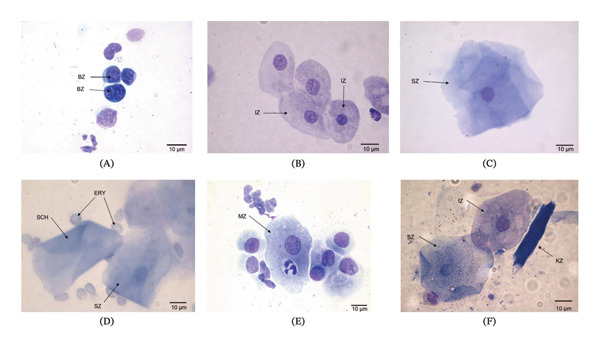
Representative vaginal cytology findings across different stages of the estrous cycle. (A) Basal cells (scale bar = 10 μm; BZ = basal cell). (B) Intermediate cells (scale bar = 10 μm; IZ = intermediate cell). (C) Superficial cell with a pyknotic nucleus (scale bar = 10 μm; SZ = superficial cell). (D) Anuclear squamous cell (cornified cell) and superficial cell, with erythrocytes in the background (scale bar = 10 μm; SZ = superficial cell; SCH = anuclear squamous cell; ERY = erythrocyte). (E) Early diestrus cell surrounded by parabasal and intermediate cells (scale bar = 10 μm; MZ = early diestrus cell). (F) Highly keratinized end‐stage superficial epithelial cell surrounded by intermediate and superficial cells (scale bar = 10 μm; KZ = highly keratinized end‐stage morphologic variants of superficial epithelial cells; IZ = intermediate cell; SZ = superficial cell).

In addition to epithelial cell classification (Figure 1), including the identification of specific structures such as early diestrus cells (Figure 1E), the number of neutrophilic granulocytes was determined. In Experiment 2, additional parameters were assessed, including the quantity of erythrocytes; cellular distribution patterns; overall cellularity per microscopic field; the presence of bacteria, secretions, and detritus; the occurrence of cotton fibers; and cytolytic changes characterized by free nuclei and cells with curled cell margins. Overall cellularity and cellular distribution patterns were assessed at 100 × magnification in at least four representative microscopic fields per smear. For quantitative comparison, 30 epithelial cells per smear were systematically identified using a meander‐shaped scanning pattern, counted, and morphometrically analyzed using image analysis software. The presence of bacteria was recorded qualitatively as either present or absent. When detected, bacterial abundance was semiquantitatively graded as low, moderate, or high. The same semiquantitative grading system was applied to cotton fibers, secretions, and detritus; free nuclei lacking a cytoplasmic rim; and epithelial cells (beyond the routinely classified vaginal epithelial cell types) showing rolled‐up or inwardly folded cell margins.

### 2.9. Statistical Analysis

Data management and statistical analyses were performed using BMDP/Dynamic software (Release 8.1; BMDP Statistical Software, Inc). Quantitative variables were assessed for normality and log‐transformed where appropriate. Descriptive statistics were calculated to summarize classification outcomes across the different stages of the estrous cycle and are presented as frequencies and percentages for categorical variables. Classification accuracy was evaluated by comparing paired cytological assessments obtained under different methodological conditions, including sampling site (vestibulum vs. cranial vagina), swab type (dry vs. saline‐moistened), and fixation method (air‐dried vs. spray‐fixed). For each comparison, agreement between paired observations was analyzed using the two‐sided McNemar test, as the data consisted of dichotomous outcomes derived from matched samples. In addition, cellular arrangement was morphologically classified into four predefined distribution patterns: isolated cells, small cell clusters, beginning nest formation, and distinct nest formation. Concordance and deviations in paired dry and saline‐moistened smears were also statistically analyzed using the two‐sided McNemar test (test of symmetry). Statistical significance was defined a priori as *p* ≤ 0.05.

## 3. Results

### 3.1. Effect of Sampling Site (Vestibulum vs. Cranial Vagina) on Vaginal Cytology

Paired cytological samples from 120 bitches were evaluated to assess cell type distribution across the vestibulum and cranial vagina. The analysis revealed distinct differences between the two sampling sites. Basal cells were rarely detected in vestibular smears, whereas they were more frequently observed in vaginal smears, consistent with their expected anatomical localization. Similarly, anuclear superficial cells (squames) were predominantly observed in vaginal smears, while KZ cells and intermediate cells were significantly more abundant in vestibular samples (*p* < 0.0001 for KZ and intermediate cells; *p* = 0.0078 for basal cells). Superficial cells were present at both sites in the majority of bitches, although notable variation existed between individual animals. Clusters (squames) and parabasal cells also demonstrated site‐dependent distribution patterns, with vestibular samples generally exhibiting higher proportions of parabasal and intermediate cells. These findings demonstrate the significant influence of sampling location on cytological composition and the interpretation of vaginal smears. The observed differences support the notion that the vestibulum may preferentially retain KZ and intermediate cells, whereas the cranial vagina reflects basal and anuclear superficial populations more accurately. This spatial variation is clinically relevant for estrous cycle staging, as it may influence the accuracy and reproducibility of cytological assessments. Overall, these results underscore the importance of standardizing the sampling site to ensure reliable evaluation of cell types, particularly for distinguishing proestrus from estrus in clinical and research settings. When cytological findings were assigned to different cycle stages, discrepancies between sampling sites were observed exclusively during estrus (Table 2). All vaginal smears obtained from dogs in estrus (*n* = 46) exhibited a characteristic estrus cytological pattern, whereas nearly half of the corresponding paired vestibular smears (*n* = 20) still showed a proestrous pattern.

**TABLE 2:** Number of smear pairs (*n* = 120) with and without agreement regarding their cytological classification into the respective stages of the cycle.

	**Agreement vestibulo–vagina (*n*)**	**No agreement (*n*)**	**Overall (*n*)**

Proestrus	19	0	19
Estrus	26	20	46
Early diestrus	32	0	32
Anestrus	23	0	23
Overall	100	20	120

### 3.2. Effect of Swab Condition (Dry vs. Saline‐Moistened) on Vaginal Cytology

**FIGURE 2:**
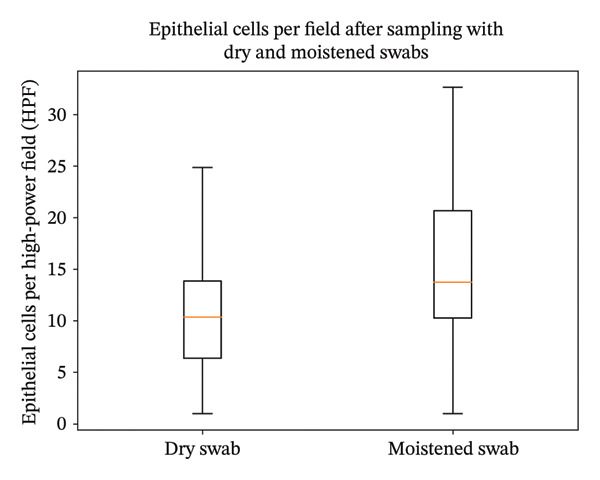
Epithelial cells per high‐power field after sampling with dry and saline‐moistened swabs (*n* = 106). Box‐and‐whisker plot illustrating epithelial cell counts per high‐power field (HPF) in vaginal cytology samples obtained using dry and saline‐moistened swabs. Moistened swabs yielded significantly higher cellularity compared to dry swabs (*p* < 0.0001). HPF, high‐power field.

Paired samples were collected from a total of 106 bitches, with each pair consisting of one smear collected using a dry cotton swab and one collected using a saline‐moistened swab. All smears were evaluable. Swab condition significantly affected smear quality and cellular presentation, although it had minimal impact on cycle stage classification. Smears obtained with saline‐moistened swabs exhibited a significantly higher cellular yield per microscopic field (*p* < 0.0001, Figure 2). These findings suggest that while saline‐moistened swabs enhance cellular recovery and smear density, they do not substantially affect the diagnostic interpretation of the estrous stage.

Bacteria, cellular debris, and secretory material were observed significantly less in moistened smears (*p* < 0.0001), whereas free nuclei were detected more often (*p* < 0.0001). Cotton fiber artifacts occurred significantly more frequently in smears collected with dry swabs (*p* < 0.0001). Cellular arrangement also differed between sampling methods: cells appeared predominantly isolated in dry swab smears, while cluster formation was more common in moistened preparations (*p* = 0.003). Among the paired cytological samples, 74/106 (70%) showed an identical distribution pattern, whereas 32/106 (30%) exhibited differences between dry and saline‐moistened preparations. In dry swab smears, cells appeared as isolated cells in 63/106 (59%), in small clusters in 13/106 (12%), with beginning nest formation in 19/106 (18%), and with distinct nest formation in 11/106 (10%). In saline‐moistened preparations, the corresponding frequencies were 49/106 (46%), 12/106 (11%), 29/106 (27%), and 16/106 (15%), respectively. Thus, isolated cells were more frequently observed in dry swab smears, whereas nest formation occurred more commonly in moistened preparations. Despite these morphological differences, concordance in estrous cycle stage classification between paired dry and moistened smears was high. Overall, 98% of paired samples were assigned to the same estrous cycle stage (Table 3).

**TABLE 3:** Number of smear pairs (*n* = 106) with and without agreement following classification into the respective stages of the cycle.

	**Agreement dry–moist (*n*)**	**No agreement (*n*)**	**Overall (*n*)**

Proestrus	25	1	26
Estrus	46	1	47
Early diestrus	19	0	19
Anestrus	14	0	14
Overall	104	2	106

### 3.3. Effect of the Fixation Method (Air‐Drying vs. Spray Fixation) on Vaginal Cytology

Paired cytological samples were collected from 106 bitches and were all evaluable. The fixation method influenced certain morphological features but did not affect overall cytological stage classification. No significant differences were observed in total cellular yield between air‐dried and spray‐fixed smears. However, air‐dried preparations contained more bacteria and secretory material than spray‐fixed smears (*p* = 0.0005), and cells with curled cell margins were more frequently observed in nonspray‐fixed smears (*p* = 0.0011, Table 4). Despite these morphological differences, 99% of paired smears subjected to either air‐drying or spray fixation yielded identical cytological cycle stage classifications. These results suggest that while fixation can alter certain cytological details, it does not compromise the accuracy of estrous stage determination, highlighting the robustness of the cytological evaluation method across different fixation protocols.

**TABLE 4:** Number of smear pairs (*n* = 106) after spray fixation and air‐drying, with and without agreement according to their classification into the respective stages of the cycle.

	**Agreement spray–air-drying (*n*)**	**No agreement (*n*)**	**Overall (*n*)**

Proestrus	36	0	36
Estrus	45	1	46
Early diestrus	12	0	12
Anestrus	12	0	12
Overall	105	1	106

## 4. Discussion

The present study was designed to systematically evaluate the methodological factors influencing the diagnostic reliability and reproducibility of vaginal cytology in bitches. Given the widespread application of vaginal cytology in canine reproductive management, especially for estrous cycle staging and breeding planning, the establishment of standardized and evidence‐based protocols is of considerable clinical relevance. Vaginal cytology is widely regarded as a rapid, cost‐effective, and minimally invasive diagnostic method that can be applied with minimal stress to the animal. Numerous authors have emphasized its value in routine clinical practice, particularly when combined with clinical examination and, where available, endocrine diagnostics [6, 10, 12–16]. More recent studies have expanded the diagnostic scope of vaginal cytology beyond reproductive staging, further underscoring the importance of reliable and standardized cytological evaluation [7, 17]. In this context, Kao et al. [13] demonstrated the diagnostic utility of vaginal swab cytology for identifying neoplastic alterations of the lower urinary tract in dogs. Their findings highlight that cytological interpretation is not limited to physiological reproductive changes but may also reveal clinically relevant pathological conditions. This broader diagnostic applicability underscores the necessity for high methodological and interpretative accuracy, as misclassification may have implications extending beyond reproductive management. In the present study, all major epithelial cell types were reliably identified at both sampling sites. However, significant site‐dependent differences in their relative abundance were observed, particularly for intermediate, parabasal, and KZ cells, which were more prevalent in vestibular smears.

The vaginal mucosa represents a hormone‐responsive mucosal epithelium, whereas the vestibular mucosa constitutes a transitional zone toward cutaneous tissue. Consequently, estrogen‐induced epithelial changes are more pronounced in the vagina, while the vestibular epithelium exhibits a weaker endocrine response. Although vestibular cytology provides reliable information for general cycle monitoring, it is inferior to vaginal cytology for precise determination of the optimal breeding time, as estrogen‐dependent cytological changes are less distinct in the vestibular epithelium. Dry swab preparations contained significantly higher amounts of bacteria, secretions, and detritus than smears obtained with moistened swabs. A plausible explanation is a dilution and flushing effect produced by the isotonic saline solution applied to the swab tip, resulting in a cleaner smear background. Another relevant aspect concerned cotton fiber contamination. The present study demonstrates that the use of moistened cotton swabs markedly reduced the presence of cotton fibers compared to dry swabs. Importantly, however, cotton fibers did not negatively affect cytological cycle stage interpretation. A significantly higher number of free nuclei lacking a cytoplasmic rim was observed in smears obtained with moistened swabs. This finding contradicts earlier assumptions that moist swabs better preserve cellular integrity in equine endometrial cytology [18]. Although free nuclei were detected significantly more frequently in saline‐moistened smears than in dry swab preparations, the strong positive concordance between paired samples suggests that individual animal‐related factors may also substantially influence this parameter. Previous authors attributed cellular deformation primarily to mechanical factors during smear preparation, particularly excessive pressure during rolling [19, 20], rather than to swab characteristics. Cellular arrangement differed consistently between sampling methods. Cells in moistened swab smears more frequently appeared in clusters, whereas dry swab preparations predominantly showed isolated cells. This pattern is likely related to the significantly higher cellular yield obtained with moistened swabs, which facilitates cluster formation. Britton [19] proposed that cautious smear rolling promotes cell clustering in mares; however, the present findings indicate, for the first time, that both the rolling technique and the swab condition contribute to cellular distribution patterns. Despite these morphological and background differences, the diagnostic outcome remained unaffected. Of the 106 bitches examined, 104 (98%) showed concordant cytological cycle stage classification in paired dry and moistened swab samples. Thus, for estrous stage determination, the choice between dry and moistened swabs is diagnostically irrelevant. Swab condition primarily influences smear quality, background cleanliness, and cellular presentation rather than the reliability of cycle stage assessment.

In the present study, paired vaginal smears from 106 bitches were used to compare fixation with a cytological spray (Merckofix, Merck) and air‐drying. With respect to the quantity of different epithelial cell types, no differences were detected between paired smears. This result was expected, as spray fixation stabilizes cells already present on the slide but does not influence the sampling process itself. In contrast, secretions and bacteria were observed in greater amounts in air‐dried smears than in spray‐fixed preparations. One possible explanation is a dilution or displacement effect caused by the application of the cytological spray, resulting in a reduced visible amount of background material. For routine cytological cycle staging, this difference is not diagnostically relevant. Overall, 105 of 106 bitches (99%) showed identical cytological cycle stage classification in paired smears, regardless of the fixation method. Therefore, for estrous stage determination, fixation with cytological spray is not essential when smears are stored for only a few days prior to staining. Short‐term air‐drying represents a diagnostically reliable and practice‐oriented alternative.

## 5. Conclusion

The results of the present study provide compelling evidence that vestibular cytology in the bitch constitutes a practical and reliable alternative to conventional vaginal cytology. Samples collection can be performed with minimal manipulation and without instrumentation, while accurately reflecting the cyclic reproductive changes. However, vestibular cytology does not provide sufficient sensitivity for the precise determination of the optimal breeding time, as estrogen‐dependent cytological alterations are considerably less pronounced than in the vaginal epithelium. Furthermore, the use of moistened swabs significantly enhances sample quality by increasing cellular yield and producing a cleaner background with reduced quantities of bacteria, debris, secretions, and cotton fibers compared to dry swab preparations. Although fixation with cytological spray is not strictly required for shorter storage periods, it may still be beneficial when longer storage or delayed evaluation is anticipated.

## Author Contributions

Katrin von Plato: data curation, investigation, methodology, writing–original draft, writing–review and editing, and software. Sait Sendag: data curation, validation, writing–original draft, and writing–review and editing. Mehmet Yildiz: validation and writing–review and editing. Axel Wehrend: conceptualization, data curation, validation, visualization, writing–review and editing, and supervision.

## Funding

No funding was received for this study.

Open Access funding enabled and organized by Projekt DEAL.

## Disclosure

All authors also reviewed and approved the final version of the manuscript.

## Conflicts of Interest

The authors declare no conflicts of interest.

## Data Availability

The data that support the findings of this study are available on request from the corresponding author.
